# Decreased Resting Functional Connectivity after Traumatic Brain Injury in the Rat

**DOI:** 10.1371/journal.pone.0095280

**Published:** 2014-04-18

**Authors:** Asht Mangal Mishra, Xiaoxiao Bai, Basavaraju G. Sanganahalli, Stephen G. Waxman, Olena Shatillo, Olli Grohn, Fahmeed Hyder, Asla Pitkänen, Hal Blumenfeld

**Affiliations:** 1 Department of Neurobiology, Yale University School of Medicine, New Haven, Connecticut, United States of America; 2 Department of Neurosurgery, Yale University School of Medicine, New Haven, Connecticut, United States of America; 3 Department of Diagnostic Radiology, Yale University School of Medicine, New Haven, Connecticut, United States of America; 4 Department of Biomedical Engineering, Yale University School of Medicine, New Haven, Connecticut, United States of America; 5 Core Center for Quantitative Neuroscience with Magnetic Resonance, Yale University, New Haven, Connecticut, United States of America; 6 Center for Neuroscience and Regeneration Research, West Haven, Connecticut, United States of America; 7 Biomedical NMR research group, Biomedical Imaging Unit, University of Eastern Finland, Kuopio, Finland; 8 Department of Neurobiology, A. I. Virtanen Institute of Molecular Sciences, University of Eastern Finland, Kuopio, Finland; 9 Department of Neurology, Kuopio University Hospital, Kuopio, Finland; University of California, Riverside, United States of America

## Abstract

Traumatic brain injury (TBI) contributes to about 10% of acquired epilepsy. Even though the mechanisms of post-traumatic epileptogenesis are poorly known, a disruption of neuronal networks predisposing to altered neuronal synchrony remains a viable candidate mechanism. We tested a hypothesis that resting state BOLD-fMRI functional connectivity can reveal network abnormalities in brain regions that are connected to the lesioned cortex, and that these changes associate with functional impairment, particularly epileptogenesis. TBI was induced using lateral fluid-percussion injury in seven adult male Sprague-Dawley rats followed by functional imaging at 9.4T 4 months later. As controls we used six sham-operated animals that underwent all surgical operations but were not injured. Electroencephalogram (EEG)-functional magnetic resonance imaging (fMRI) was performed to measure resting functional connectivity. A week after functional imaging, rats were implanted with bipolar skull electrodes. After recovery, rats underwent pentyleneterazol (PTZ) seizure-susceptibility test under EEG. For image analysis, four pairs of regions of interests were analyzed in each hemisphere: ipsilateral and contralateral frontal and parietal cortex, hippocampus, and thalamus. High-pass and low-pass filters were applied to functional imaging data. Group statistics comparing injured and sham-operated rats and correlations over time between each region were calculated. In the end, rats were perfused for histology. None of the rats had epileptiform discharges during functional imaging. PTZ-test, however revealed increased seizure susceptibility in injured rats as compared to controls. Group statistics revealed decreased connectivity between the ipsilateral and contralateral parietal cortex and between the parietal cortex and hippocampus on the side of injury as compared to sham-operated animals. Injured animals also had abnormal negative connectivity between the ipsilateral and contralateral parietal cortex and other regions. Our data provide the first evidence on abnormal functional connectivity after experimental TBI assessed with resting state BOLD-fMRI.

## Introduction

Traumatic brain injury (TBI) is a common brain insult as every 21 seconds, one person in the USA sustains a TBI, and it is estimated that TBI annually affects about 1.7 million Americans (http://www.cdc.gov/injury/about/focus-tbi.html). White matter and axonal injury are common pathologic sequela of TBI [1]. Importantly, injury-related breakdown of the neuronal networks associates with a wide spectrum of functional impairments, including compromised somatomotor, cognitive, and behavioral performance [1]. Based on previous studies, network abnormalities apparently also contribute to the development of other co-morbidities, particularly post-traumatic epilepsy (PTE) [2]. Assessment of the development, severity, and progression of abnormalities in connectivity between the brain regions following TBI could pinpoint the at-risk patients for functional deficits, and guide targeting of therapeutic interventions for those who benefit most.

Blood oxygenation level dependent (BOLD) functional magnetic resonance imaging (fMRI) has enabled the mapping of different brain networks in health and disease [3], [4]. BOLD-fMRI at rest measures remotely distributed brain networks fluctuating synchronously in the brain. Resting functional connectivity was first used to reveal motor brain networks [5]. For more than 15 years, resting functional connectivity has been used to study different neuronal networks such as those involved in motor, sensory, visual, memory, language, or cognitive processing [6], [7] Recently it has also been used to study epilepsy in humans [8]–[11] as brain regions involved in BOLD-fMRI during seizures can be used as seed regions for analysis of resting functional connectivity [8], [10], [12]. So far, resting state BOLD-fMRI has not been applied in experimental models of TBI, even though it could be expected to reveal network abnormalities not easily detectable with other methodologies *in vivo*.

We hypothesize that local cortical damage caused by TBI results in a widespread disruption of cortical and subcortical networks, which associates with functional impairments, particularly the development of increased seizure susceptibility. The consequent PTE develops as progressive fragmentation of functional and structural connectivity. Therefore, resting BOLD-fMRI could provide us clues about changes in neuronal networks that are critical for post-traumatic epileptogenesis. To address these hypotheses, we assessed network changes by resting BOLD-fMRI in rats, in which post-traumatic epileptogenesis was induced with lateral fluid-percussion injury (FPI), an established model of human PTE [13], [14]. We focused on brain areas known to be injured by the impact and recently suggested to be involved in post-traumatic epileptogenesis, including the parietal cortex, hippocampus, and thalamus [15]–[17]. Our goal was to find a noninvasive method for assessment of the network changes during post-TBI epileptogenesis that could ultimately be applied to studies of human PTE.

## Material and Methods

### Animals

Adult male Sprague-Dawley rats (Harlan Netherlands B.V., Horst, the Netherlands) were used. The animals were housed in individual cages under standard conditions (cage size 53. 32.5. 20 cm, 12 h light/12 h dark rhythm, lights off at 7 p.m., room temperature 21±1°C, and humidity 50–60%). Water and food were available *ad libitum*. Procedures related to the induction of TBI were approved by the Animal Ethics Committee of the Provincial Government of Southern Finland, and carried out in accordance with the guidelines of the European Community Council Directives 86/609/EEC. All experimental procedures were in full compliance with Yale University Institutional Animal Care and Use Committee protocols approved in agreement with the National Institutes of Health.

### Induction of lateral fluid-percussion brain injury

TBI was induced by lateral fluid-percussion injury (FPI) as described previously [13], [18]. Briefly, rats were anesthetized with intraperitoneal injection (i.p.) of a cocktail, containing sodium pentobarbital (582 mg/kg), chloral hydrate (60 mg/kg), magnesium sulphate (127 mg/kg), propyleneglycol (43%), and ethanol (11.7%). Then, they were inserted into a stereotaxic frame with lambda and bregma at the same horizontal level. A midline scalp incision was made and a 5-mm circular piece of parietal bone was removed over the left cortex, midway between the lambda and bregma and midway between the sagittal suture and temporal ridge. Care was taken to leave the dura intact. A plastic female Luer-Lock connector was secured in the craniotomy with Vetbond adhesive (3M, St. Paul, MN, USA). The connector was anchored with dental acrylate to a screw placed in the skull rostral to the bregma. Animals were placed on heating pads while anesthetized to maintain the normothermic temperature. Ninety minutes (90 min) after injection of anesthetic, the rat was attached to the fluid percussion device (AmScien Instruments, Richmond, Virginia, USA) to produce TBI (pressure level 3.4±0.01 atm). Animals were removed from the device, and thereafter, dental cement, screw, and Luer-Lock connector were removed, and scalp was sutured. Sham-operated animals underwent surgery but were not injured. Mean weight of animals at the time of TBI or sham-operation was 350±11 g.

### Survival BOLD–fMRI

Survival MRI was performed at four months post-TBI. On each imaging day we scanned a pair of animals, that is, a sham-operated rat was scanned in the morning and a rat with lateral FPI in the afternoon and *vice versa* (randomly counterbalanced) (between 9:00 am and 6:00 pm). For imaging, rats were positioned prone in a specially designed plastic holder with the head fixed and bregma positioned at the center of the quadrature coil. The animal was then inserted into the magnet with its head positioned at the isocenter of the magnet. Electroencephalogram (EEG) was recorded simultaneously with BOLD–fMRI, using a pair of 1-mm diameter carbon-filament electrodes (WPI, Sarasota, FL). The purpose of the EEG recording during the MRI was to detect epileptiform activity that would interfere with the ‘resting state’ needed for the MRI analysis. To minimize MRI signal distortion, the carbon filaments were placed between the scalp and the surface of the skull in the frontal and occipital areas and secured to the skin with tissue glue (3M Vetbond, 3M Animal Care Products, MN) [19]. The exposed portion of each electrode crossed the midline from left to right in the coronal plane. The EEG signals were acquired in differential mode between the two electrodes, amplified (×100), and filtered (1–30 Hz) using a Model 79D Data Recording System (Grass Instruments Co., Quincy, MA). EEG signals were digitized and recorded (sampling rate 1 KHz) using a CED Micro 1401 and Spike 2 software (Cambridge Electronic Design, Cambridge, UK). All MRI data were acquired using a 9.4 T Bruker horizontal bore (16–cm internal diameter) spectrometer (Agilent Technologies), equipped with passively shielded shim/gradient coils (47.5 G/cm) operating at 400.5 MHz for protons. A quadrature (1×2) coil with two 2.1 cm loops was used as transmitter and receiver. To optimize the homogeneity of the static magnetic field, the system was shimmed before each experiment using global manual shimming. 3% isoflurane in air/oxygen (70%/30%) was used as induction anesthesia. Thereafter, rats received a bolus of dexmedetomidine (i.p., 0.3 mg/kg; Domitor, Pfizer, Karlsruhe, Germany) and isoflurane was discontinued after 5 min. At 15 min after bolus injection, a continuous infusion of dexmedetomidine (0.1 mg/kg/h; 1 ml/h, i.p.) was started. During subsequent animal preparation and MRI imaging, animals were breathing spontaneously on room air. Rectal temperature was monitored and maintained at ∼37.5°C by a water-circulated heating pad (Model # TP3E, Gaymar Industries, Inc., NY). At the end of the MRI experiments, dexmedetomidine was antagonized by atipamezole (0.1 mg/kg, i.p.; Antisedan, Pfizer, Karlsruhe, Germany).

Anatomical images for each animal were acquired with 12 interlaced slices in the coronal plane using fast spin echo multi slice, with repetition time (TR) 4000 ms; echo time (TE) 48 ms; flip angle  = 40–55°; field of view (FOV) 25.6×25.6 mm; 256×256 matrix size; in–plane resolution of 98×98 µm; and slice thickness 1000 µm, without gap. BOLD–fMRI data were obtained in the same planes as anatomical images. We used single-shot spin echo echo planar imaging (SE–EPI). SE–EPI data were acquired with the following parameters: TR, 2000 ms, TE, 34 ms; excitation flip angle 90°; inversion flip angle 180°; FOV, 25.6×25.6 mm, 64×64 matrix size; in-plane resolution, 390×390 µm; and slice thickness 1000 µm. All SE–EPI experiments were acquired with 12 slices. The 12 slices were acquired over 2000 ms, followed by a 2s pause before the next image onset so that EEG could readily be interpreted during data acquisition. Time between onset of consecutive image acquisitions was therefore 4s. We acquired 150 images per experimental run, resulting in a total imaging time of 600 s for one experimental run and four runs were acquired per animal. Sixteen dummy scans occurred before the receiver was turned on. Dummy scans were used to ensure that the proton spin system was in a steady state before data were collected.

### EEG recording

#### Electrode implantation

A week after survival MRI (i.e., 4.5 months after lateral FPI), sham-operated and injured rats were anesthetized with ketamine (100 mg/kg), xylazine (10 mg/kg), and acepromazine (1 mg/kg), and then, inserted in a stereotactic frame (David Kopf Instruments, Tujunga, CA) for implantation of stainless steel electrodes (Part # MS333/3-A, tripolar, uncut untwisted 0.005; Plastics One Inc., Roanoke, VA; internal control # 8LMS3333XXXE, pedestal height: 8 mm). To provide a good electrical contact before wrapping around skull screws, the polyimide insulation was scraped off from the ends of the recording electrodes, exposing stainless steel wire up to 10 mm from the tip but leaving insulation intact proximally, as verified under the microscope. Small burr holes [using Micro Drill Steel Burrs, 2.3 mm shaft diameter, 44 mm overall length; item # 19007-14, Fine Science Tools (USA), Inc.] were made in the skull without damaging the dura and electrodes were secured to the skull using stainless steel screws (Plastics One, Part # 0-80X1/16, internal control # 8L010121201F with shaft length = 1.60 mm, head diameter = 2.50 mm, shaft diameter = 1.57 mm). EEG recording electrodes were implanted in the ipsilateral (left) frontal (AP +2.0, ML +2.0 mm) and contralateral parietal cortex (AP -6.0, ML +2.0 mm). A ground electrode was placed in the midline over the cerebellum. An anchoring screw without electrode was placed contralateral to craniotomy at an equal distance (ML -2.0 mm) between the coronal suture and the lambdoidal suture. Dental acrylic (Cat # 1255710; Henry Schein Inc, Indianapolis, IN; Lang Jet Denture Repair Acylic) was used to fix the electrode pedestal to the skull. Level of anesthesia was monitored by respiration, heart rate, glabrous skin perfusion, and responses to foot pinch.

#### EEG monitoring

One week after recovery from electrode implantation, awake and freely-moving sham-operated and injured rats underwent a 1-h baseline EEG recording (between 9:00 a.m. and 6:00 p.m). Recordings were done via a tripolar cable (Plastics One, Inc., Catalog# 335-340-3 0-SPR 80CMtripolar) and a commutator (Plastics One, Inc., Catalog# 8BSL3CXCOMMT) connected to a Grass CP 511 amplifier (Grass- Telefactor, Astro Med, Inc., West Warwick, RI). Band pass frequency filter settings were 1–300 Hz. Signals were digitized at a sampling rate of 1 KHz with an NI USB-6008 A/D converter and LabView 7.1 software (National Instruments, Austin, TX), and analyzed using Spike 2 (Cambridge Electronic Design, Cambridge, UK).

### Pentylenetetrazol (PTZ) test

Right after the baseline EEG monitoring, sham-operated and injured rats were exposed to a PTZ test to assess seizure susceptibility as described before [13]. Briefly, PTZ (1,5-pentamethylenetetrazol, Santa Cruz Biotechnology, Inc, USA) was dissolved in sterile 0.9% sodium chloride (final concentration of 12.5 mg PTZ/ml 0.9% sodium chloride), and administered at a dose of 25 mg/kg (i.p.). Each rat received a single injection of PTZ solution. Following the PTZ injection, rats were placed separately into cages where they could move freely, and EEG was recorded for 60 minutes after PTZ administration. Behavior was monitored by an observer. The time of the occurrence of epileptiform behavioral events was recorded and scored according to a modified Racine's scale (1  =  twitching, freezing, 2  =  myoclonic jerks of one forelimb; 3  =  bilateral forelimb clonus; 4  =  forelimb clonus with rearing; 5  =  tonic–clonic convulsion).

### Analysis of EEG data

EEG signals acquired during survival EEG–fMRI were first processed using Spike 2 software and magnetic field-induced artifacts were reduced as described previously [19]–[21]. Thereafter, EEGs were screened for occurrence of electrographic interictal epileptiform discharges (IEDs) in EEG acquired during fMRI as well as before and after PTZ administration. IED was defined as a high–amplitude rhythmic discharge containing a burst of slow waves, spike-wave and/or polyspike-wave components and lasting >1 s.

After PTZ administration, latency to the first IED was calculated. Start and end times of all IEDs were marked in the EEG files manually using Spike 2 software and a script provided by CED (Cambridge, U.K.). Intervals containing artifact were also marked and excluded from the analysis. Number of IEDs was calculated during the 60 min after PTZ administration. Based on the evolution of IEDs seen after PTZ injection, we divided this 60 min in three 20 min time segments, 1). 0–20 min, 2). 21–40 min, and 3). 41–60 min. Then we performed paired t-test between IEDs in these three 20 minutes time segments in FPBI and in sham rats.

### Histologic analysis

#### Fixation and cryoprotection

One hour after the PTZ-test, rats were deeply anesthetized with sodium pentobarbital (Euthasol, 100–150 mg/kg i.p.) and perfused intracardially with 0.9% sodium chloride followed by 4% cold paraformaldehyde (PFA) in 0.1 M phosphate buffered saline (PBS) (pH = 7.4). After perfusion, brains were postfixed in 4% PFA in PBS at +4 °C for 1 h. Before cryopreservation, brains were immersed in 0.02 M potassium phosphate buffered 20% glycerol at +4°C for at least 36 h. Thereafter, brains were blocked placed on dry ice for 15 minutes, and then stored at −70°C until histologic processing.

#### Tissue processing

The brains were sectioned in the coronal plane (30 µm, 1-in-5 series) with a sliding microtome. The first series of sections was stored in 10% formalin at room temperature and the remaining four series in a cryoprotectant tissue-collecting solution (30% ethylene glycol, 25% glycerol in 0.05 M sodium phosphate buffer) at −20°C until stained.

#### Histologic stainings

The first series of sections was stained for thionin to identify the cytoarchitectonic boundaries of different brain areas as well as the distribution and severity of tissue damage.

An adjacent 1-in-5 series of sections was stained for calcium using the Alizarin red method [22] to determine calcium deposition. In brief, sections were mounted on gelatinized glass slides and immersed in 2% Alizarin Red (w/v, distilled water pH 4.1 to 4.3; Merck, Darmstadt, Germany) for 30 s followed by a rinse in distilled water. Sections were quickly dehydrated with acetone and xylene and mounted in Depex (VWR International Ltd, Poole, UK).

A possible role for iron accumulation in the pathophysiological events after mild TBI has been suggested recently [23]. Therefore, to stain iron deposition in the brain, one series of sections was stained with Perls' Prussian Blue [24]. Sections were hydrated with distilled water and incubated in solution containing 1% HCl and 1% potassium hexacyanoferrate (II)-trihydrate (Merck, Germany) for 15 min at room temperature. To visualize the anatomic structures of the brain, the sections were counterstained with Mayer's hematoxylin (Sigma-Aldrich).

Myelinated fibers were stained with gold chloride solution. For staining, coronal sections were mounted on gelatinized slides and dried at 37°C. Mounted sections were incubated at room temperature in the dark for 11–14 h in a 0.2% gold chloride solution (HAuCl4.3H2O, G-4022 Sigma) made in 0.02 M sodium phosphate buffer (pH 7.4) containing 0.9% NaCl. The slides were then washed twice in 0.02 M sodium phosphate buffer in 0.09% NaCl (4 min each), and placed in a 2.5% sodium thiosulfate solution for 5 min. After 3 washes (10 min each) in the buffer solution, sections were dehydrated through an ascending series of ethanol, cleared in xylene, and cover-slipped with DePeX mounting medium (BDH, Laboratory Supplies, Dorset, UK).

### Resting BOLD-fMRI correlation analysis

Resting BOLD–fMRI connectivity analysis was performed using in-house programs written in MATLAB 7.1. Although rats were paralyzed during experiments, all fMRI series were first screened for movement artifacts using a movie function and center–of–mass analysis, restricted to voxels within the brain boundaries, to ensure that all runs exhibited movement of less than 20% of a pixel in either the x or y direction as described previously [19], [25].

**Figure 1:**
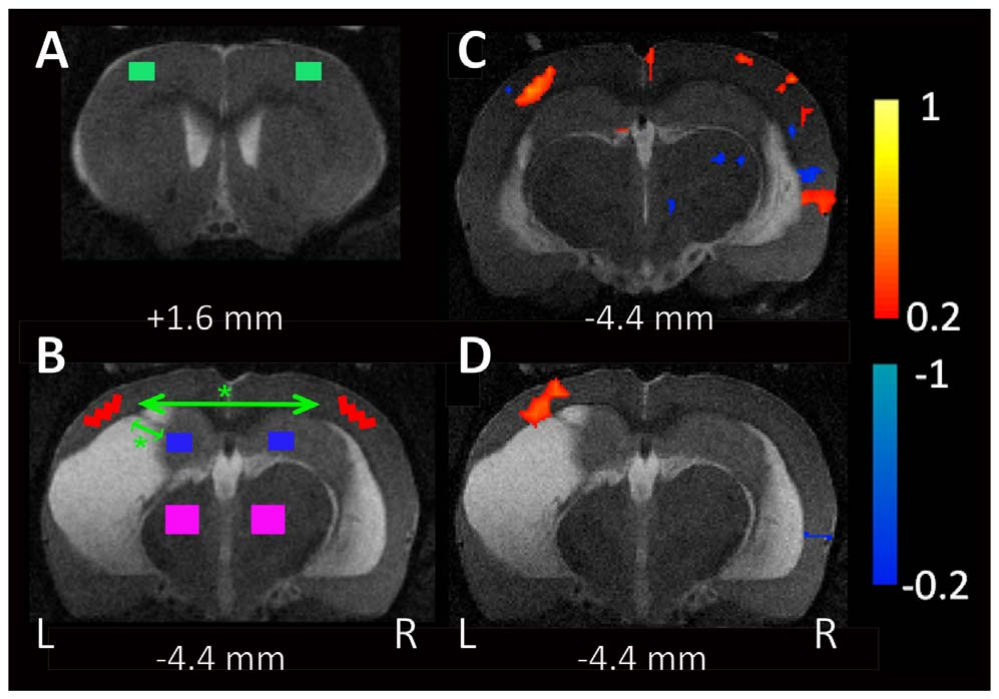
Decreased functional connectivity in TBI rats vs. controls. Two coronal MRI slices (A, B) from a rat with lateral FPI (note the tissue loss in the left hemisphere) demonstrating the brain regions of interest (ROIs) superimposed on coronal BOLD-fMRI images used for resting BOLD–fMRI signal correlation analysis. Four ROIs were made in each hemisphere (8 ROIs total): frontal cortex, parietal cortex, hippocampus, and thalamus (L, left; R, right). Panel B shows schematically using arrows the significant differences in connectivity (1) between the ipsilateral and contralateral parietal cortices and (2) between the ipsilateral parietal cortex and hippocampus in the rats with lateral FPI and sham-operation. Statistical significance: *, p<0.05. (C) Example of resting functional connectivity in a sham-operated control rat. Pearson correlation values are shown using the left parietal cortex ROI (B) correlated to the whole brain. Some positive correlation is seen with the contralateral parietal cortex and ipsilateral hippocampus. (D) Example of the same analysis for the left parietal cortex ROI in a TBI animal shows reduced connectivity. Warm colors represent increases and cool colors decreases in connectivity; scale bars are for Pearson correlation with display threshold  = 0.2. Voxels lying outside the brain or in CSF are not shown.

The data were next band-pass filtered (0.01<f<0.08 Hz) to remove low–frequency drift and reduce the influence of high–frequency noise. For fMRI analysis four pairs of regions of interests (ROIs) were made: bilateral frontal and parietal cortex, hippocampus, and thalamus (**Fig. 1A, B**).

ROIs were individually drawn on each rat's MRI data. We selected these brain regions because they are known to be involved in posttraumatic epileptogenesis [15], [17]. ROI size was 6 voxels in frontal cortex and in hippocampus, 7 voxels in parietal cortex, and 12 voxels in the thalamus. ROI size was different in different regions in order to quantitate maximum fMRI signals available from each brain regions at the same time to avoid signal from non-brain area. For individual rats, a mean time course was calculated for each ROI by averaging the time courses of all voxels within the ROI. We then computed the Pearson's correlation coefficient between mean time courses of each possible pair of ROIs. Subsequently, these correlation coefficients(r) were converted to z-scores by Fisher's z transform z(r) = 0.5 ln[(1+r)/(1−r)] [26]. To normalize for differences in number of images, each z score was divided by the square root of variance, calculated as √1/(n−3), where n is the degrees of freedom defined as the number of image acquisitions within each epoch.

For group statistical analysis, we compared z scores for each ROI pair between the sham-operated and injured rats. To accomplish this, we performed one–way ANOVA followed by Tukey's Honestly Significant Difference (HSD) method for *post hoc* pair-wise comparisons to assess the group differences.

## Results

There was no acute mortality in 8 rats randomized to the sham group in the 48 hours following sham injury. Two of 8 sham-operated animals later died before MRI. One of the 13 rats randomized to the TBI group died during anesthesia before injury. From the 12 remaining animals randomized to lateral FPI group, 3 (25%) died during the first 48 h corresponding to moderate-severe injury severity. From the remaining 9 TBI animals, 2 died before MRI. Results are therefore from 6 sham operated and 7 TBI animals. On the day of fMRI mean weights of sham operated animals (495±31 gm, mean ±SD) were not different (p = 0.63) from TBI animals (488±17 gm).

### Spontaneous epileptiform activity in EEG

Survival BOLD-fMRI was performed at 4 months after lateral FPI or sham injury. No IEDs were detected during survival fMRI runs in sham-operated or injured rats. Also, no IEDs were detected in the 1-h baseline EEG recordings performed before PTZ test. Thus the baseline EEGs obtained in the same animals approximately two weeks apart (during survival fMRI and prior to PTZ) were similar and did not show spontaneous IEDs.

### Decreased latency to the first seizure and increased number of IEDs in rats with TBI

**Figure 2:**
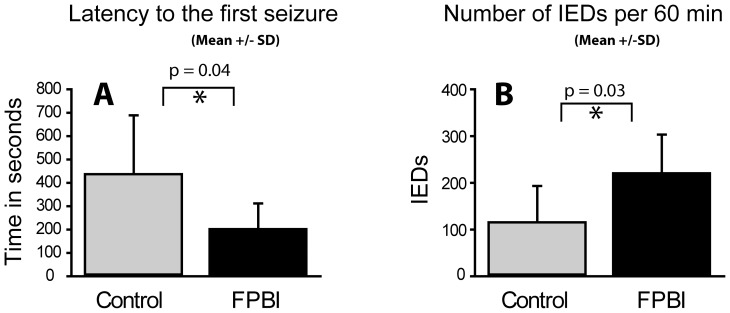
(A) Latency to the first interictal epileptiform discharge (IED) and (B) Number of IEDs in rats with lateral FPI or sham-operation. Note a decrease in latency to the 1^st^ IED (p = 0.03) and an increase in IEDs (p = 0.03) in injured rats (n = 7) as compared to sham-operated animals (n = 6). Abbreviations: SD, standard deviation.

**Figure 3:**
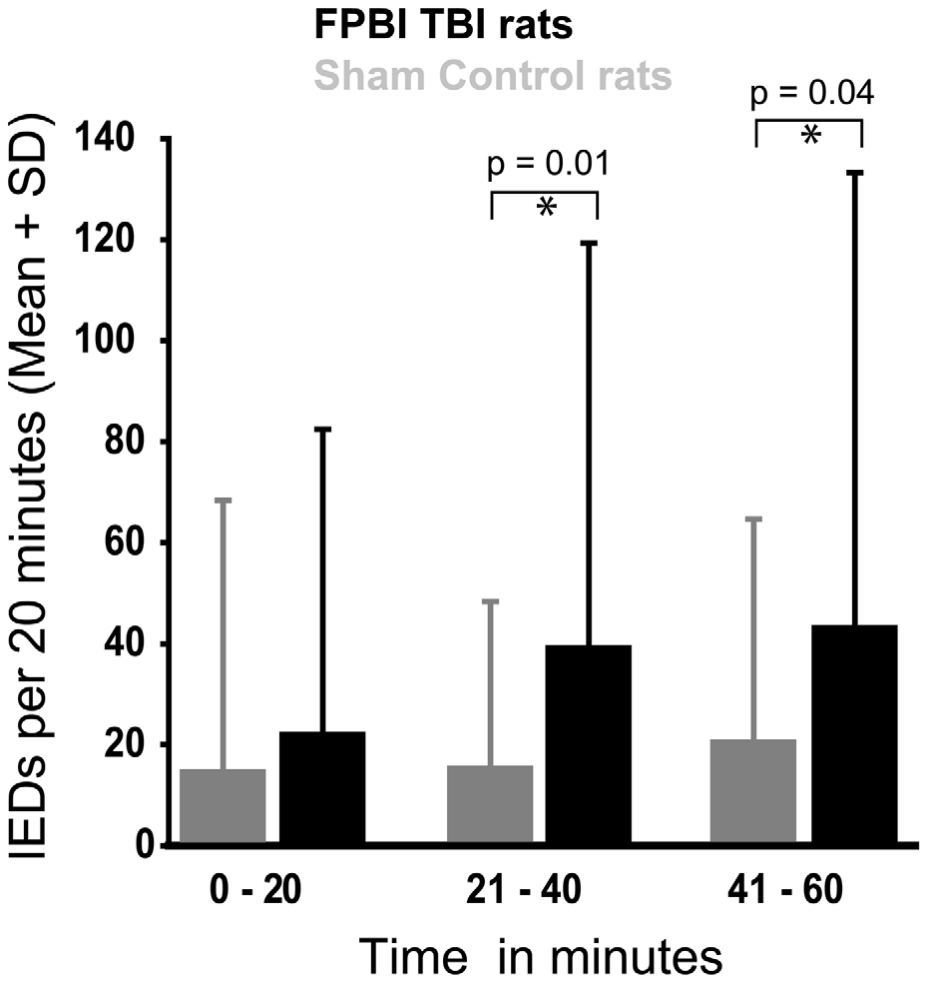
Evolution of interictal epileptiform discharges (IEDs) over time after administration of pentylenetetrazol (25 mg/kg body weight) in rats with lateral FPI (n = 7) and sham-operated animals (n = 6). Abbreviations: SD, standard deviation.

EEG recordings after PTZ administration revealed frequent IEDs in all rats. Post-PTZIEDs were associated with twitching and freezing, corresponding to a score of 1 on the modified Racine's scale. In rats with TBI, the latency to the 1^st^ IED was shorter than that in the sham-operated animals (207±40 s and 442±101 s, p = 0.04) (**Fig. 2A**). In 5 of 7 the injured rats, the latency to the 1^st^ spike was less than -1SD of the control mean. The total number of IEDs was higher in injured rats as compared to that in sham-operated animals (223±30 vs.118±31per 60 min, p = 0.03) (**Fig. 2B**). Interestingly, the frequency of IEDs in injured rats (n = 7) increased already during the first 20 min post-PTZ, becoming significant during 21–40 min (p = 0.01) and 41–60 min (p = 0.04) post-PTZ as compared to sham-operated animals (n = 6) (**Fig. 3**). In 7 of 7 rats, the total number of IEDs was higher than +1SD of the control mean.

### Decreased resting functional connectivity in rats with TBI

Rats with TBI had lower resting fMRI correlation coefficients than sham-operated rats (**Table 1**). An example of resting fMRI connectivity in a sham-operated control compared to a TBI rat is shown in Figure 1 C, D. At the group level, rats with TBI had significantly decreased correlation coefficients between the left and right parietal cortices as well as ipsilateral to injury between the parietal cortex and hippocampus as compared to sham-operated animals (**Table 1** **,** **Fig. 1B**). Injured rats also had abnormal negative connectivity values between the left and right parietal cortex and other brain regions, not seen in control animals (**Table 1**). We attempted to correlate the functional connectivity in individual animals to the seizure susceptibility with PTZ administration, however the sample size was too small to demonstrate a significant relationship (data not shown).

**Table 1:** Correlation coefficients of resting BOLD-fMRI signals and their significances from different brain regions in sham-operated rats (n = 6) and in rats with traumatic brain injury (TBI) (n = 7) and comparison between the two groups.

Significance (p values) comparing correlation coefficients from different ROIs: sham-operated rats vs. rats with TB		Frontal Cx - R	HC - R	Parietal Cx - R	Thalamus - R	Frontal Cx - L	HC – L	Parietal Cx – L
	**Thalamus - L**	0.36	0.74	0.41	0.55	0.74	0.32	0.23
	**Parietal Cx - L**	0.99	0.11	0.03	0.16	0.88	0.03	
	**HC - L**	0.99	0.57	0.06	0.41	0.26		
	**Frontal Cx - L**	0.50	0.68	0.16	0.32			
	**Thalamus - R**	0.57	0.70	0.09				
	**Parietal Cx - R**	0.30	0.17					
	**HC - R**	0.55						
**Correlation (r) values in different ROIs in sham-operated rats**	**Thalamus - L**	0.024	0.076	-0.005	0.230	0.091	0.248	0.070
	**Parietal Cx - L**	0.111	0.135	0.187	0.160	0.034	0.185	
	**HC - L**	0.044	0.114	0.106	0.199	0.017		
	**Frontal Cx - L**	0.163	0.045	0.076	0.058			
	**Thalamus- R**	0.042	0.135	0.046				
	**Parietal Cx - R**	0.106	0.174					
	**HC - R**	0.029						
**Correlation (r) values in different ROIs in rats with TBI**	**Thalamus - L**	0.119	0.050	0.074	0.186	0.123	0.192	-0.048
	**Parietal Cx - L**	0.110	0.018	0.019	0.032	0.055	-0.08	
	**HC - L**	0.045	0.065	-0.033	0.160	0.122		
	**Frontal Cx - L**	0.073	0.074	-0.024	0.149			
	**Thalamus - R**	0.098	0.101	-0.061				
	**Parietal Cx - R**	0.012	0.011					
	**HC - R**	-0.036						

**Abbreviations:** Cx, cortex; L, left; R, right. Values in **boldface** were significantly different (p<0.05) in sham-operated and injured rats.

### Histology

**Figure 4:**
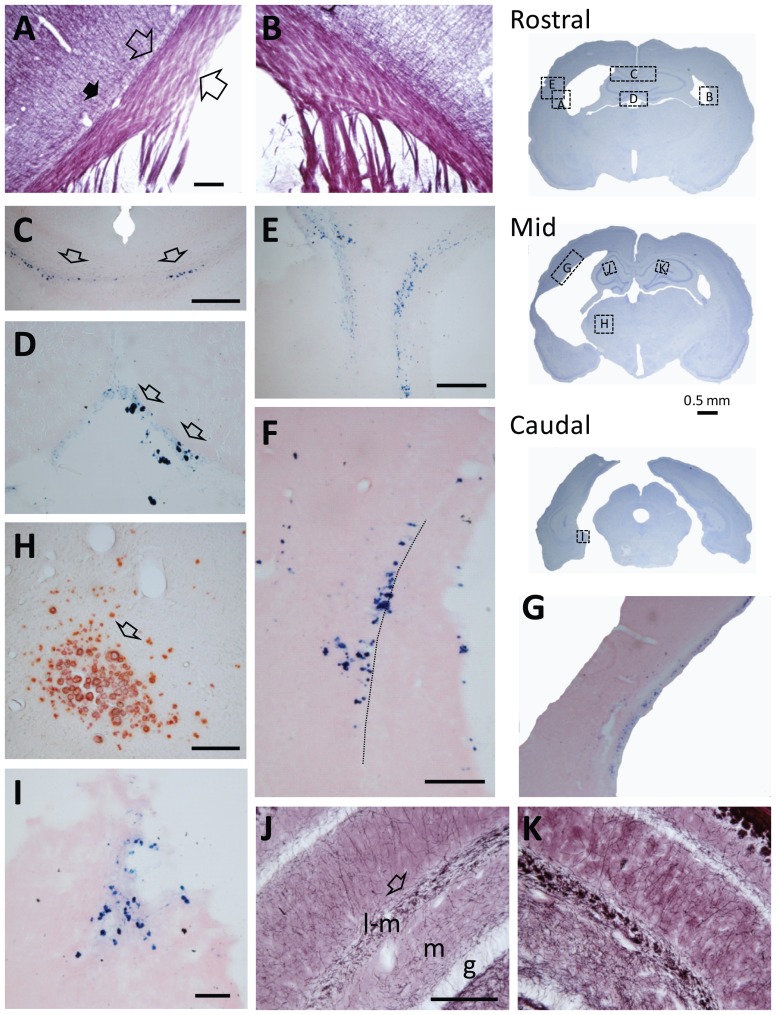
Summary of pathological findings in animals with lateral FPI. Three thionin-stained whole brain coronal sections on the right show the approximate location, from which panels A-K were taken. Rostro-caudal level of sections corresponds to the level of MRI slices (**A**) Myelin-stained section demonstrating a reduced thickness of white matter (fiber bundle between the open arrows) and in particular, reduction in the density of fibers in layer VI (black arrow) of the S1 region ipsilaterally. (**B**) Note a thicker white matter band contralaterally and abundance of myelinated fibers in layer VI. The pattern of staining is comparable to that in sham-operated controls (not shown). (**C**) Perl-stained sections showing iron deposits in corpus callosum bilaterally (open arrows). (**D**) Iron deposits in plexus choroideus (open arrows). (**E**) Iron deposits in cortical lesion and deep portion of the cortex near white matter band. (**F**) Iron deposits in the subgranular region of the dentate gyrus. Dashed line indicates the border between the granule cell layer and the hilus. (**G**) Iron deposits in subcortical white matter on the lesion side. (**H**) Alizarin-stained calcifications in the ipsilateral thalamus (open arrow). (**I**) Iron deposits in the parasubiculum. (**J**) Myelin staining from the ipsilateral hippocampus showing a reduced density of fibers in the stratum lacunosum-moleculare of the CA1 (open arrow) compared to contralateral hippocampus. Contralaterally (**K**) the pattern of staining is comparable to that in sham-operated controls (not shown). Abbreviations: g, granule cell layer; m, molecular layer; l-m, stratum lacunosum molecular of CA1; p, pyramidal cell layer. Scale bars equals in panels A–F, H–K 100 µm.

Histologic findings were qualitatively similar in all 7 injured animals available for analysis and they are summarized in **Fig. 4**. Iron deposits were present in all injured animals and were observed ipsilaterally in several white matter bands, including the external capsule at the level of the cortical lesion (**Fig. 4E, G**) and medial aspects of fimbria. Iron deposits were seen ipsilaterally in the corpus callosum above the septal end of the hippocampus in all injured rats, and they extended contralaterally in 4 of 7 animals (**Fig. 4C**). In tissue parenchyma, patches of iron deposits were present in all injured rats in the subgranular region of the infra pyramidal blade of the temporal dentate gyrus (**Fig. 4F**). In 2 of 7 injured rats we also found them in the parasubiculum (**Fig. 4I**). Occasionally they were also seen ipsilaterally in the dorsal midline thalamic area and bilaterally in the ventral brain stem.

Calcium deposits were exclusively located in the ipsilateral thalamus, most typically in the ventroposterior nucleus or in the area dorsomedial or dorsolateral to it (**Fig. 4H**).

As reported previously [27], myelin staining revealed reduced fiber density in layer VI of the perilesional cortex (**Fig. 4A**) and in stratum lacunosum moleculare of the CA1 of the ipsilateral hippocampus (**Fig 4J**) in all injured rats.

## Discussion

The present study was designed to test a hypothesis that resting state BOLD-fMRI functional connectivity can reveal network abnormalities in brain regions that are connected to the lesioned cortex, and that these changes associate with functional impairment, particularly epileptogenesis. To our knowledge these are the first data reporting abnormalities in functional connectivity in experimental TBI using resting state BOLD-fMRI. In fact, there are no previous functional connectivity studies published in human TBI either.

### A selective set of pathways show impaired functional connectivity after experimental TBI which associate with seizure susceptibility

Our previous studies have shown that up to 80% rats with moderate to severe lateral FPI develop increased seizure susceptibility, and up to 50% of the animals express spontaneous seizures in a 1-year follow-up [13], [28]. The present study confirms these observations by showing that 100% of animals with TBI induced with lateral FPI 5 months earlier had increased IEDs and 71% had a reduced latency to the 1st epileptiform spike in PTZ test, indicating increased seizure susceptibility. Analysis of resting state BOLD–fMRI functional connectivity in the same animals showed decreased connectivity between the ipsilateral and contralateral parietal cortex, between the ipsilateral parietal cortex and ipsilateral hippocampus as well as negative connectivity between the ipsilateral and contralateral parietal cortex with other regions. Moreover, histologic analysis revealed abnormalities in pathways mediating the connectivities, including corpus callosum, capsula externa, and perforant pathway. Regarding the abnormal connectivity between the ipsilateral and contralateral parietal cortex, an obvious candidate for the cause is the lesion in the corpus callosum which was present in each animal with lateral FPI. However, the explanation for an abnormal connectivity between the parietal cortex and ipsilateral hippocampus is apparently polysynaptic, and could include abnormalities in several ipsilateral white matter bands including the external capsule which were also present in all injured animals. Other physiological changes such as alterations in axonal function [29], [30] could also potentially contribute to reduced connectivity even in the absence of obvious white matter structural changes.

Desynchronization has been reported during the preictal state preceding pharmacologically-induced epileptic seizures. Under those circumstances it is characterized by a biphasic network dynamics with an early desynchronization phase followed by a late resynchronization phase, in which the activity and synchronization of the network gradually increase [31]. Though we did not directly address the possible role of early desynchronization in the development of seizures, our findings of decreased resting functional connectivity, which may be secondary to structural changes induced by the lateral FPI support the concept of dissociation of brain network synchrony during epileptogenesis [32].

A previous study in rats with lateral FPI at 8 months after brain injury showed decreased cerebral blood flow (CBF) and increased vessel density in the perilesionalcortex [17]. The hippocampus, however, showed bilateral decreases in CBF but no change in vessel density. Therefore, in addition to histologic lesions, the reduced cortical and hippocampal CBF may explain both the later seizure susceptibility and decreased connectivity of ipsilateral parietal cortex to ipsilateral hippocampus. Because ipsilateral and contralateral hypoperfusion in the cortex as well as in the hippocampus can persist for several months after lateral FPI, they could contribute an overall decrease in resting functional connectivity observed in the present study [15].

In addition to epileptogenesis, impaired functional connectivity could contribute to other post-TBI co-morbidities. The different stages of working memory have been associated with poor interhemispheric coherence on EEG in the frontal and temporal regions in patients with mild TBI [33]. In moderate TBI patients, Gupta and colleagues [34] have shown that impaired structural connectivity measured through diffusion tensor imaging (DTI) fractional anisotropy and radial diffusivity correlated with neuropsychological test scores. Follow-up upon recovery in these DTI indices were associated with recovery in neurocognitive deficits [34]. Our findings provide evidence for bilateral network abnormalities in TBI in brain regions which are necessary for working memory.

### Methodological considerations

Our study was performed under anesthesia, while posttraumatic epileptogenesis studies using fMRI in human PTE patients could be done in the awake state. Throughout all imaging sessions, anesthesia dosage was kept constant, and a continuous rate of infusion of dexmedetomidine anesthesia greatly enhances our ability to obtain stable and reliable MRI measurements in the rodent model [35]–[37], without blocking primary somatosensory cortex-evoked potentials [38]. Although ideally the measurements should be repeated in the awake state, the differences between groups are unlikely to be caused entirely by anesthesia. Awake MRI experiments are feasible in rodent models, but considerably more technically challenging, since it is necessary to train and habituate each animal for a prolonged period [39], [40].

The resting functional connectivity approach offers a number of advantages in studying chronic changes in posttraumatic epileptogenesis. The relatively slow time scale of neurovascular events measured by resting BOLD–fMRI connectivity provides a window into disease mechanisms over longer time scales. In contrast to EEG, resting BOLD–fMRI allows the detection of signals in the whole brain simultaneously.

Furthermore, resting state BOLD–fMRI can be used to assess basal functional connectivity in both superficial and deep brain networks by calculating temporal correlations of BOLD–fMRI signals between remote brain areas during the resting state [5], [41]. This method has been shown to provide enough sensitivity to reveal altered functional connectivity in language networks in temporal lobe epilepsy [10] and also in generalized spike-wave epilepsy [8], [9]. Our BOLD–fMRI findings expand these data by showing that after TBI there is a dissociation of short-range as well as long-range brain network connectivity in injured rats with increased seizure susceptibility. In further work with a larger sample size it may be possible to investigate relationships between seizure susceptibility and resting functional connectivity in individual animals.

## Conclusion

We found a decreased connectivity between the ipsilateral and contralateral parietal cortex and between the parietal cortex and hippocampus on the side of injury in rats with lateral FPI as compared to sham-operated animals. We also found abnormal negative connectivity in rats with TBI between the ipsilateral and contralateral parietal cortex and other regions. Our study using resting state BOLD-fMRI functional connectivity provides the first proof-of-concept evidence that this methodology can be applied for identification of mechanisms and biomarkers for posttraumatic epileptogenesis, which can be used to monitor the progression of epileptogenesis and antiepileptogenic efficacy of treatments.
