# Correction: Decreased Resting Functional Connectivity after Traumatic Brain Injury in the Rat

**DOI:** 10.1371/journal.pone.0105899

**Published:** 2014-08-14

**Authors:** The PLOS ONE Staff

Table 1 is missing boldface formatting due to errors in the typesetting process. The correct version of Table 1 can be viewed below.

**Table 1:** Correlation coefficients of resting BOLD-fMRI signals and their significances from different brain regions in sham-operated rats (n  =  6) and in rats with traumatic brain injury (TBI)(n  =  7) and comparison between the two groups.

		Frontal Cx - R	HC - R	Parietal Cx - R	Thalamus - R	Frontal Cx - L	HC – L	Parietal Cx – L
Significance (p values) comparing correlation coefficients from different ROIs: sham-operated rats vs. rats with TBI	**Thalamus - L**	0.36	0.74	0.41	0.55	0.74	0.32	0.23
	**Parietal Cx - L**	0.99	0.11	**0.03**	0.16	0.88	**0.03**	
	**HC - L**	0.99	0.57	0.06	0.41	0.26		
	**Frontal Cx - L**	0.50	0.68	0.16	0.32			
	**Thalamus - R**	0.57	0.70	0.09				
	**Parietal Cx - R**	0.30	0.17					
	**HC - R**	0.55						
Correlation (r) values in different ROIs in sham-operated rats	**Thalamus - L**	0.024	0.076	-0.005	0.230	0.091	0.248	0.070
	**Parietal Cx - L**	0.111	0.135	**0.187**	0.160	0.034	**0.185**	
	**HC - L**	0.044	0.114	0.106	0.199	0.017		
	**Frontal Cx - L**	0.163	0.045	0.076	0.058			
	**Thalamus- R**	0.042	0.135	0.046				
	**Parietal Cx - R**	0.106	0.174					
	**HC - R**	0.029						
Correlation (r) values in different ROIs in rats with TBI	**Thalamus - L**	0.119	0.050	0.074	0.186	0.123	0.192	-0.048
	**Parietal Cx - L**	0.110	0.018	**0.019**	0.032	0.055	**-0.08**	
	**HC - L**	0.045	0.065	-0.033	0.160	0.122		
	**Frontal Cx - L**	0.073	0.074	-0.024	0.149			
	**Thalamus - R**	0.098	0.101	-0.061				
	**Parietal Cx - R**	0.012	0.011					
	**HC - R**	-0.036						

**Abbreviations:** Cx, cortex; L, left; R, right. Values in **boldface** were significantly different (p<0.05) in sham-operated and injured rats.

In the Materials and Methods section under “Analysis of EEG Data,” the last line of the second paragraph incorrectly refers to FPBI. This should state “FPI” instead.

Figure 2 and Figure 3 incorrectly refer to the experimental data as “FPBI.” This should state “FPI.” The authors have provided corrected versions of Figure 2 and Figure 3 below.

**Figure 2:**
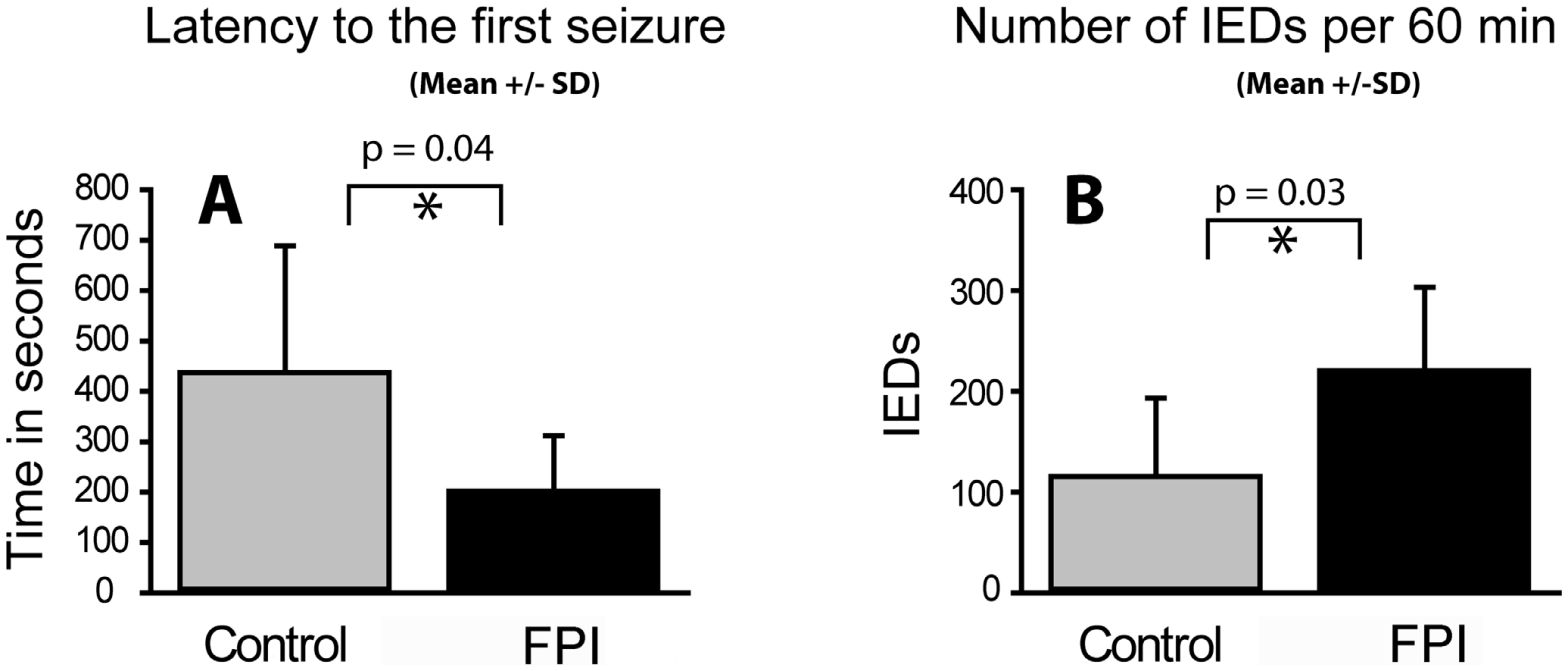
(A) Latency to the first interictal epileptiform discharge (IED) and (B) Number of IEDs in rats with lateral FPI or sham-operation. Note a decrease in latency to the 1^st^ IED (p  =  0.03) and an increase in IEDs (p  =  0.03) in injured rats (n  =  7) as compared to sham-operated animals (n  =  6). Abbreviations: SD, standard deviation.

**Figure 3:**
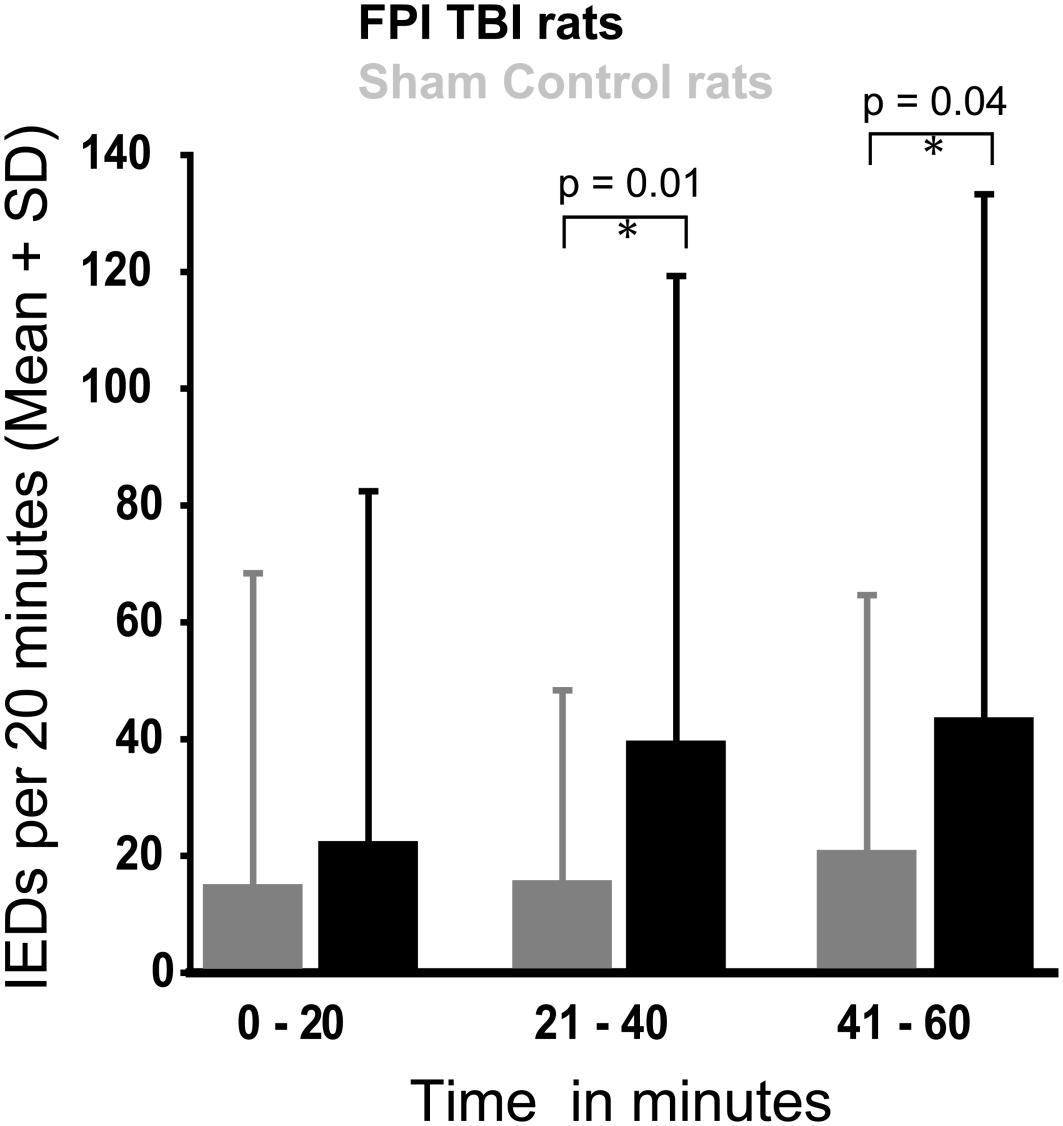
Evolution of interictal epileptiform discharges (IEDs) over time after administration of pentylenetetrazol (25 mg/kg body weight) in rats with lateral FPI (n  =  7) and sham-operated animals (n  =  6). Abbreviations: SD, standard deviation.
